# The Effects of Music Therapy-Singing Group on Quality of Life and Affect of Persons With Dementia: A Randomized Controlled Trial

**DOI:** 10.3389/fmed.2018.00279

**Published:** 2018-10-15

**Authors:** Heeyoun Kim Cho

**Affiliations:** Department of Music Therapy, Temple University, Philadelphia, PA, United States

**Keywords:** music therapy, dementia, singing, listening, quality of life, affect

## Abstract

Dementia is a clinical syndrome that is progressive and degenerative, affecting memory, behavior, emotion, and personality. Persons with dementia often experience deterioration of cognitive ability, as well as various behavioral and psychological disturbances, which significantly contribute to reduced quality of life and emotional well-being. The demand for long-term care continues to rise rapidly and it is therefore critical to develop effective strategies and evidence-based interventions to improve the quality of life for persons with dementia. Music therapy has drawn attention as a promising non-pharmacological approach for persons with dementia. A variety of music interventions including singing and listening to music have been widely applied for dementia care not only by music therapists, but also by other healthcare professionals. There are, however, little research studies that compare possible effects of music therapy interventions with those of music-based approaches on dementia care. The purpose of the current study was to compare the short-term effects of a music therapy-singing group with those of a music medicine-listening group and a control-TV group, on quality of life and affect of persons with dementia at a long-term care facility. The music therapy-singing group was facilitated by a music therapist, whereas the music medicine-listening and the control-TV group were led by nursing home activity staff. Fifty-two participants, whose ages range from 67 to 99 years old, were randomly assigned to one of the three groups, and 37 participants completed the interventions. The participants in each group were engaged for a 40-min session twice a week for four consecutive weeks. Quality of life was measured at the baseline and after the last session and only the music therapy-singing group demonstrated significant improvements when compared to the other groups. Positive and negative affect were measured at three points, including pre and post the first, fourth and eighth sessions. Only the music therapy-singing group significantly increased positive affect scores and decreased negative affect scores. The findings of the current study suggest that music therapy with active group singing may be an effective non-pharmacological intervention in improving quality of life and affect of persons with dementia at long-term care settings.

**Clinical Trial Registration**: http://www.germanctr.de/drks_web/, registration number DRKS00014934.

## Introduction

Dementia is a clinical syndrome that is progressive and degenerative, affecting memory, thinking, behavior, emotion and personality, and currently no treatment is available to cure or alter the course of dementia (1). The prevalence of dementia increases dramatically with age (2) and the behavioral and psychological symptoms of dementia contribute significantly to a decreased sense of well-being and quality of life of persons with dementia (3). Behavioral symptoms, as well as an inability to perform daily activities and social functions due to cognitive impairment, are the leading reasons that persons with dementia and their families seek long-term care services (4). Consequently, the pervasiveness of dementia in long-term care settings is much higher than in the community, imposing considerable challenges on the healthcare providers and it is critical to develop effective strategies and evidence-based interventions to improve quality of life for persons with dementia in those settings (5).

Music therapy has gained an increasing attention as a promising non-pharmacological approach in dementia care (6). Music therapy is “a systematic process of intervention wherein the therapist helps the client to promote health, using music experiences and the relationships that develop through them as dynamic forces of change” (7). Music therapy studies in dementia care have utilized a variety of music interventions, such as singing, playing musical instruments, listening, or improvising, with an emphasis on the therapeutic relationship and a process. Music interventions have been widely applied not only by music therapists, but also by other healthcare professionals in dementia care. Compared to music therapy studies that utilize extensively live and active music methods by a credentialed music therapy professional, music medicine interventions are characterized by the use of primarily passive music listening implemented by medical personnel (8). Both music therapy and music medicine studies in the existing literature have reported positive effects of music interventions on various aspects of dementia care, including behavioral symptoms, cognitive skills, social and emotional functions, motor performances and physiological changes (6, 9, 10). However, the evidence of music therapy and music medicine interventions in dementia care is inconclusive, due to poor quality and methodological limitations of most music-related studies (10).

Quality of life is a concept that is concerned with a person's emotional and physical well-being, interpersonal relations, personal development, self-determination, and social inclusion (11) and has been increasingly recognized as a valuable outcome measure of dementia care (3). An assessment of dementia-specific quality of life in residential settings, such as long-term care facilities, can identify any unmet needs of persons of dementia and can be used to enhance their quality of life, which is a high priority of any healthcare providers (12). Quality of life is strongly influenced by the individual's perception of mood, a sense of control, an interaction with the environment and a relationship with others (13), as well as by affect, especially the experience of positive emotions (14). Recent research studies have examined the effects of music or music therapy interventions on quality of life of persons with dementia and the findings are not consistent (15–20).

Among a variety of music interventions for persons with dementia in music therapy and music medicine studies, singing and music listening have been utilized most frequently. Singing has been widely used for persons with dementia for various therapeutic outcomes, due to its capacity for social, emotional, cognitive, and physical engagement with a relatively low threshold for participation (21–24). In music therapy studies for persons with dementia, singing was utilized either as a primary intervention (18, 25–28) or with other interventions, such as music listening, instruments playing, rhythm activities, improvisation, or movement (20, 29–32).

Singing has been also adopted by professional caregivers at long-term care settings especially during daily care routines for persons with dementia (33–35). When professional caregivers with no formal music training sang to persons with dementia during daily care routines, the usual reactions of aggression, combativeness, and confusion were replaced by a sense of understanding and an enhanced communication. Furthermore, singing by professional caregivers during care routines alleviated distress not only in care receivers, but also in caregivers, who reported considerable improvement in their perceptions of the situations (36).

Music listening has been also utilized for persons with dementia in various levels. A receptive music listening has been extensively adopted by medical personnel for dementia care in the long-term care settings. In most music medicine studies, persons with dementia are often exposed to listening primarily to pre-recorded music (37–41) and the effects of different characteristics of the music conditions were evaluated, such as individualized music vs. classical (38) or soothing music vs. folk songs vs. popular music (41). The music listening experiences of persons with dementia in most music medicine studies are naturally passive without requiring much responses or interaction with the facilitator.

As evidenced in the existing literature, singing and listening have been applied in dementia care in various ways by music therapists and other healthcare professionals. However, studies that compare the effects of singing with those of listening are lacking. Furthermore, any possible differences between music interventions by music therapist professionals and those by other healthcare professionals in dementia care have not been investigated and need to be tested and defined. The results of such a study would be of a great value to music programming for dementia care.

The framework of the current music therapy-singing intervention was influenced by a person-centered care model that emphasizes the importance of affirming and maintaining the personhood of individuals with dementia through communications and interpersonal relationships (42). Recognizing the identity of persons with dementia and finding ways to maintain and develop their unique selfhood through interactions and communications are fundamental components of the person-centered care model (43, 44). The person-centered approach encourages caregivers to focus less on what is done and more on how it is done, honoring the individual's choices and needs (42) and is found to be highly correlated with quality of life of persons with dementia in long-term care settings (45). Based on the person-centered philosophy, the music therapy-singing group of the current study was designed and implemented with the therapeutic goals of promoting a sense of self-worth, belonging, and accomplishment.

The purpose of the current study was to compare the possible effects of a music therapy-singing group on the quality of life and affect of persons with mild, moderate, and severe dementia living in a long-term care facility with those of a music medicine-listening group and a control-TV group.

## Methods

### Participants

The participants were recruited from a Veterans' Home in upstate New York, which is a long-term care skilled nursing facility with a total of 242-beds capacity. The residents of the facility who met specified criteria were invited to the current study. The inclusion criteria included residents with a documented diagnosis of dementia, who were between 65 and 100 years of age, had no significant hearing impairment, and were able to sit in a chair or wheelchair for at least 1 h. Residents with severe psychiatric conditions, as well as receptive or expressive language problems, were excluded.

### Research design

The study was a randomized controlled trial with a pretest-posttest design adopting three groups, including two music interventions and a control group. Using a randomization number table, participants were randomly assigned to one of the three groups, including a music therapy-singing group (MT), a music medicine-listening group (MM), or a control-TV group (TV).

### Procedure

The protocol of the present study was reviewed and approved by Temple University's Institutional Review Board (IRB) for the Protection of Human Subjects. Due to the absence of IRB at the facility where the research study was to be conducted, the facility agreed to rely on Temple University's IRB for review and continuing oversight of the current research study.

After obtaining IRB approval, residents' charts were reviewed by the researcher and an activity staff to identify potential participants based on the inclusion and exclusion criteria of the study. Once potential participants were identified, an invitation to the study was sent out to the potential participants or family members of the participants who were not able to make decisions themselves with an explanation of the study, including the purpose, procedures, risks, benefits, confidentiality, and subjects' rights.

**Figure 1:**
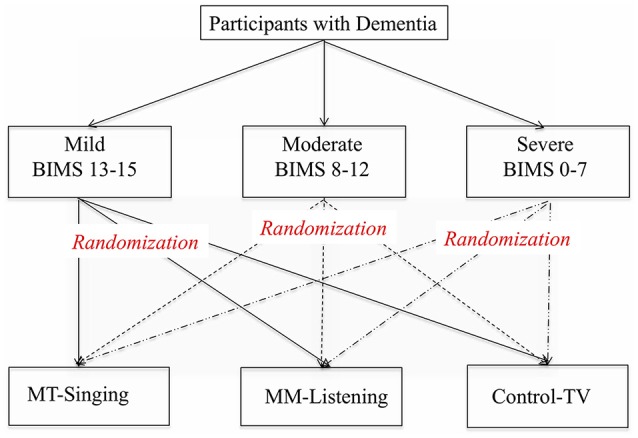
Randomized allocation process.

Once acquiring consent forms, the participants were divided into three sub-groups based on their cognitive status following Brief Interview for Mental Status (BIMS) scores, including mild (BIMS score 13–15), moderate (BIMS score 8–12) and severe (BIMS score 0–7). The total BIMS score provides a reliable estimate of an individual's cognitive function and mental capacity (46) and the facility where the study was to be conducted routinely administered this instrument every 6 months unless significant changes were detected. A list of participants in each cognitive category was created by an activity staff who was also responsible for identifying potential participants via a chart review at the beginning of the current study. For the random assignment, the list of participants was given to another activity staff with specially assigned numbers in place of the participants' names. The participants' names were not revealed to the activity staff who was responsible for the random assignment until the randomization process was completed in order to ensure the allocation concealment. The participants in each group of cognitive status were randomly assigned to three intervention groups, including a music therapy-singing group (MT), a music medicine-listening group (MM), and a control-TV group, utilizing a random number table from a statistical text book (47) (See Figure 1).

### Intervention

Once the participants for each group were identified, an intervention delivery schedule was developed. Due to the large number of participants assigned to each group, the three intervention groups, including a music therapy-singing group (MT), a music medicine-listening group (MM), and a control group-TV (TV), were divided into two sub-groups, one group held in the morning and another held in the afternoon. The participants in each group were engaged for a 40-min session twice a week for four consecutive weeks.

#### Music therapy-singing group (MT)

A total of eight music therapy-singing programs were created and implemented by the researcher of the current study, who is a Board-Certified Music Therapist with 15 years of experience in dementia care. She was not involved in recruitment, randomization of participants or data collection. The eight lists of songs for the music therapy-singing groups were developed by the researcher, reflecting the participants' reported preferences. Participants' preferred songs, music genres and musicians were assessed through open-end questionnaires, when the consent forms to participate in the study were collected. Each session consisted of different sets of songs, except the greeting and good-bye songs. Group singing was facilitated and accompanied by the therapist on a Yamaha keyboard and the song sheets with lyrics were provided to the participants as needed. Based on the person-centered care model, the music therapy-singing group was implemented with an emphasis on promoting a sense of self-worth, belonging and accomplishment through spontaneous expression and mutual interaction. The singing group was partly protocolized, following the pre-determined sequence of songs for each session, to ensure the consistency of the content with those of the music listening group. The sequence of songs was carefully determined by the therapist so that the participants may experience a combination of stimulation and relaxation. However, the therapist's interaction with the participants was not protocolized or limited so that any verbal or musical responses of the participants during singing can be processed. Each song was repeated twice. However, instead of singing through the songs straightforward, the therapist paused or repeated parts of the songs as needed to validate and develop the participants' musical strengths, as well as to enhance the participants' contribution to the musical interaction. The therapist also varied the speed or volume of the accompaniment on the keyboard to stimulate the participants' interest and to maximize their musical experience, following a natural flow of increase and release of a musical tension.

#### Music medicine-listening group (MM)

The music listening group was facilitated by activity staff and the participants of this group were engaged in listening to a CD which contained the identical songs in the same order that were used for the music therapy-singing group. The researcher created eight CDs using the pre-recorded songs from the iTunes. When several recordings by various musicians were available, the recordings by the participants' preferred musician were selected. The activity staff who were assigned to lead the music listening group were instructed to facilitate the group in the same manner as usual activity programs and to validate and process the participants' responses, if any.

#### Control-TV group (TV)

A control-TV group was facilitated by activity staff and the participants in this group watched a DVD featuring an episode of the comedy program, “I Love Lucy” for a similar duration as the singing and the listening groups. The activity staff who were assigned to lead the TV group were instructed to facilitate the group in the same manner as usual activity programs and to validate and process the participants' responses, if any.

### Outcome measures

In order to assess the effects of each of the three interventions, two standardized measurement tools were utilized; the Quality of Life-Alzheimer's Disease (QOL-AD) (12, 48) and the Positive and Negative Affect Schedule (PANAS) (49). Activity staff who were not involved with providing the treatment interventions interviewed participants using these measurement tools.

#### The quality of life-alzheimer's disease (QOL-AD)

**Figure 2:**
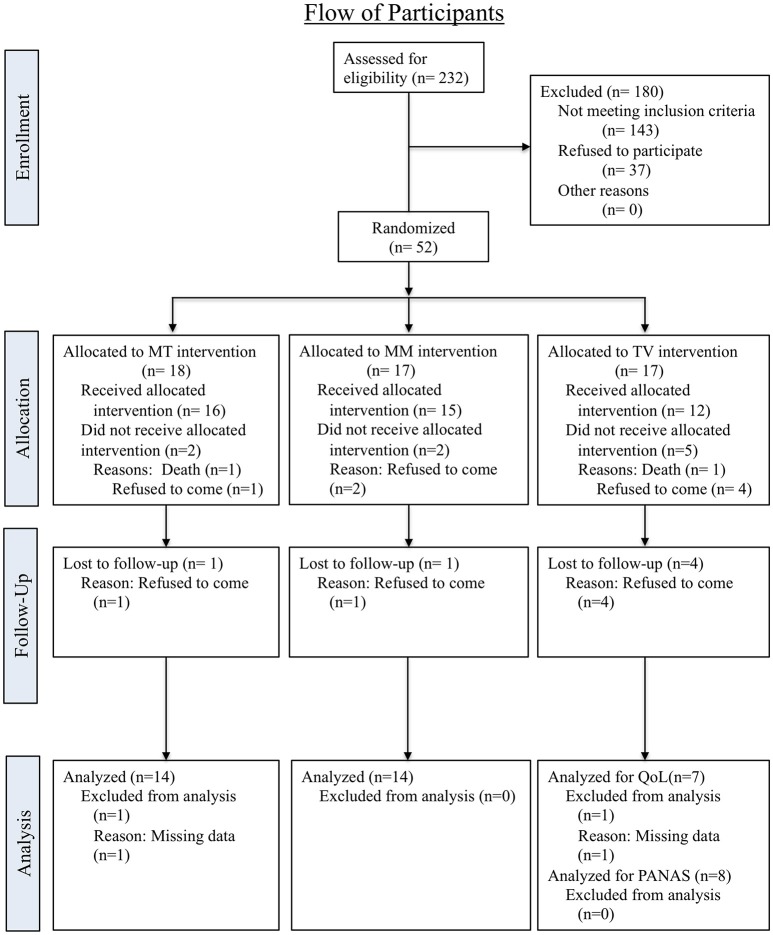
Consort flowchart for participant recruitment and allocation.

This measurement instrument is designed to assess the quality of life in older adults with cognitive impairment. The original measure had 13 items (48), but was modified specifically for use in long-term care settings to include 15 items (12). The questionnaire evaluates one's physical condition, mood, interpersonal relationships, and ability to participate in meaningful activities. Each item is rated on a 4-point scale ranging from “1 = poor” to “4 = excellent,” resulting in a single mean score ranging from 15 to 60, with higher score indicating greater QOL. Utilizing this assessment tool, quality of life was evaluated twice, once before the first intervention session and once after the last intervention session (See Figure 2). This instrument has two versions, one designed directly for persons with dementia, and the other for caregivers. For the current study, quality of life was assessed directly from persons with dementia via interviews conducted by activity staff.

#### The positive and negative affect schedule (PANAS)

This 20-item scale measures the two primary dimensions of mood, both negative and positive affects (49). Participants of each group were asked by activity staff to complete the PANAS Questionnaire immediately before and after the first, fourth and eighth session.

### Training

The present study was conducted by a primary researcher, who is a Board-Certified Music Therapist with 15 years of experience in dementia care and assisted by the facility's 12 activity staff throughout the research process. The researcher provided a training session for the activity staff, who were informed regarding the protocol and their roles for the current research study, including recruiting participants, obtaining informed consents, transporting participants to the location of assigned intervention sessions, providing interventions for a music medicine-listening group and a control-TV group, and collecting data. Their roles were not overlapped. Those who were assigned to collect data also received an additional instruction regarding utilizing the outcome measurement tools, the QOL-AD (12) and the PANAS (49). These activity staff's experiences in dementia care ranged from 10 to 29 years, and their routine responsibilities at the facility included providing a variety of activities, as well as conducting a periodic assessment, evaluation, and documentation. Therefore, their roles specifically assigned for the current study were not much different from their existing routine duties.

## Results

### Data analysis

The Statistical Package for the Social Sciences (SPSS) version 22.0 (SPSS Inc., Chicago, IL, USA) was used for data analysis. Prior to analysis, basic frequencies were run on the data to screen for missing values and outliers and to establish data entry accuracy. Data were analyzed using a repeated-measures analysis of variance (ANOVA) to determine specific effects of the interventions.

### Demographic data

Eligible participants were recruited from a local long-term care facility in September 2015. At the time of the recruitment, a total of 232 residents lived at the facility. Eighty-nine residents, who met the criteria of the study, were invited to the study. Of the 89 eligible participants, 52 (58%) agreed to participate and 37 (42%) refused. Fifty-two participants were randomly assigned to one of three groups, including a music therapy-singing group, a music medicine-listening group and a control-TV group. Of the 18 participants who were randomly assigned to the music therapy-singing group, 15 (83.3%) completed the intervention and data from 14 participants only were included for analysis due to missing data for both quality of life and affect. Seventeen participants were assigned to the music medicine-listening group and 14 (82.4%) completed the intervention and were analyzed for both quality of life and affect. Of the 17 participants who were assigned to the control-TV group, only eight (47.1%) completed the intervention. For the quality of life measurement, only seven of eight who completed a TV intervention were analyzed due to missing data. For PANAS (positive and negative affect scale), data from all of eight participants in the TV group were analyzed. Details of recruitment, allocation and participation for each group are included in Figure 2.

All of the three groups included both male (*n* = 43) and female (*n* = 9) participants in three different cognitive statuses. All participants were Caucasians. The participants in the MT-singing group were between the ages 67 to 99 years with a mean age of 85.06, while those in the MM-listening group ranged from 75 to 98 years with a mean age of 87.94. The control-TV group included participants aged from 74 to 97 with a mean age of 87.00. See Table 1 for the detailed baseline demographic and clinical characteristics of participants in each group.

**Table 1:** Baseline demographic and clinical characteristics.

**Variable**	**Total *N* of participants**	**MT-singing group**	**MM-listening group**	**TV group**
**GENDER**				
Male	43	15	14	14
Female	9	3	3	3
Total	52	18	17	17
**AGE:**				
Mean		85.06	87.94	87.00
Standard Deviation		8.71	5.91	5.97
**BIMS:**				
Mean	1.90	10.22	10.24	9.88
Standard Deviation	0.82	4.36	3.99	3.55
**MENTAL STATUS:**				
1 (BIMS 13-15)	20	7	7	6
2 (BIMS 8-12)	17	6	5	6
3 (BIMS 0-7)	15	5	5	5

*N, number of participants; BIMS, Brief Interview for Mental Status*.

To determine if there were any differences regarding gender among groups, chi-square analysis was conducted and the results showed that there was no difference among groups regarding gender, Chi Square = 0.008, *p* = 0.999. A one-way ANOVA was also conducted and there was no difference on age, *F*_(2, 49)_ = 0.772, *p* = 0.468, or on cognitive status, *F*_(2, 49)_ = 0.043, *p* = 0.958.

### Music preferences

Regarding the participants' preferred type of music, old country western was the most preferred type of music identified by the participants, followed by big band, classical, rock & roll, patriotic, jazz, musical, polka, pop, hymn, Dixie land, folk and rag music. The favorite musicians identified by the participants included Bing Crosby, Dean Martin, Elvis Presley, Gene Autry, George Strait, Hank Williams, Johnny Cash, Kate Smith, Roy Rogers, Lawrence Welk, and Kenny Rogers. A list of the favorite songs identified by the participants is displayed in Table 2.

**Table 2:** A List of favorite songs reported by participants.

**FAVORITE SONGS OF PARTICIPANTS**		
Allegheny moon Alley cat America the beautiful Anchors away Anything you can do Are you lonesome tonight At last Autumn leaves Battle hymn of the republic Beer barrel polka Bell bottom trousers Blue moon Blue moon of Kentucky By the light of the silvery moon Bye bye blackbird Carolina moon Come fly with me Coming in on a wing and a prayer Danny boy Don't be cruel Don't sit under the apple tree East of sun west of moon Eight days a week Embraceable you Everybody loves somebody Folsom Prison Blues Fugue for tinhorns Get me to the church on time God bless America God bless U.S.A. Going to Kansas City Good night my someone Green green grass of home Greensleeves	Half as much Happy trails Hey good looking Home on the range How great thou art I can't give you anything but love I could have danced all night I left my heart in San Francisco I walk the line If I had a hammer In the cool, cool, cool of the evening I'll be in Scotland before you I'll be with you in apple blossom time It's only a paper moon Jambalaya King of the road Lili Marlene Lollipop Lucille Mack the knife Make the world go away Mairzy doats Moon over Miami Moonlight bay Moonlight serenade My blue heaven New York, New York Off we go into the wild blue yonder Oh how I hate to get up in the morning Oh what a beautiful morning Old Cape Cod Old man river Pennsylvania Polka Pittsburgh, Pennsylvania Praise the Lord and pass the ammunition	Rhapsody in blue Red river valley Ring of fire Rose of Texas Sentimental Journey Seventy six trombones Shine on harvest moon Shoo fly pie and apple pan dowdy Side by side Sioux city Sue Sixteen tons Sophisticated lady Stardust Sunday morning coming down Swinging on a star Tennessee waltz That's amore The caissons go rolling along The marines hymn There's a tear in my beer There's no business like show business This is the army, Mr. Jones To each his own Under the boardwalk What do you do in the infantry What's he doing in my world When I'm sixty-four When Johnny comes marching home When the moon comes over the mountain White cliffs of Dover Yellow rose of Texas You can't roller skate in a buffalo herd You gave me a mountain Your cheating heart

### Quality of life analysis

The effects of two music treatment groups and a control group on quality of life were measured before the first session, and after the last session, via a direct interview conducted by activity staff with the participants, utilizing a standardized questionnaire scale, QOL-AD (12). Out of 52 participants who were randomly assigned to three intervention groups, 37 (71.2%) completed the interventions and 35 (67.3%) were included in the final analysis, due to missing data. The means and standard deviations are presented in Table 3.

**Table 3:** Means and standard deviations of QOL-AD.

**QOL-AD**	**Group**	**Mean**	**Standard deviation**	***N***
**PRE-FIRST SESSION**				
	MT	38.71	4.23	14
	MM	39.29	8.15	14
	TV	40.71	6.55	7
	Total	39.34	6.35	35
**POST-EIGHTH SESSION**				
	MT	47.29	6.58	14
	MM	41.43	7.09	14
	TV	45.71	6.37	7
	Total	44.63	7.09	35

*N, number of participants; MT, music therapy; MM, music medicine*.

To determine the possible effects of a music therapy-singing, a music medicine-listening, or a control-TV group on quality of life of persons with dementia, data were analyzed using a repeated-measures analysis of variance. The analysis revealed that there was a significant difference between the pretest and posttest, *F*_(1, 32)_ = 27.19, *p* < 0.05, ηp^2^ = 0.459, and a significant interaction, *F*_(2, 32)_ = 4.56, *p* < 0.05, ηp^2^ = 0.222, as seen in Table 4.

**Table 4:** Repeated measures ANOVA for quality of life.

**Source**	**Sum of squares**	**df**	**Mean square**	***F***	***P***	**ηp2**
Group	124.20	2	62.10	0.862	0.432	0.051
Error between	2304.29	32	72.01			
Pre-Post	432.14	1	432.14	27.19	0.001^*^	0.459
Interaction	145.00	2	72.50	4.56	0.018^*^	0.222
Error within	508.57	32	15.89			

* *p < 0.05*.

To follow up on the significant interaction, a simple effects analysis was conducted and determined that there were significant effects of a music therapy-singing group on quality of life of persons with dementia (*t* = 7.02, *p* = 0.001) and only the singing group significantly increased quality of life between the pre-test and the post-test. The effects of a music medicine-listening group (*t* = 1.39, *p* = 0.187) or those of a control-TV group (*t* = 1.82, *p* = 0.118) on quality of life were not significant. The three groups did not differ significantly at either pre-test or post-test.

**Figure 3:**
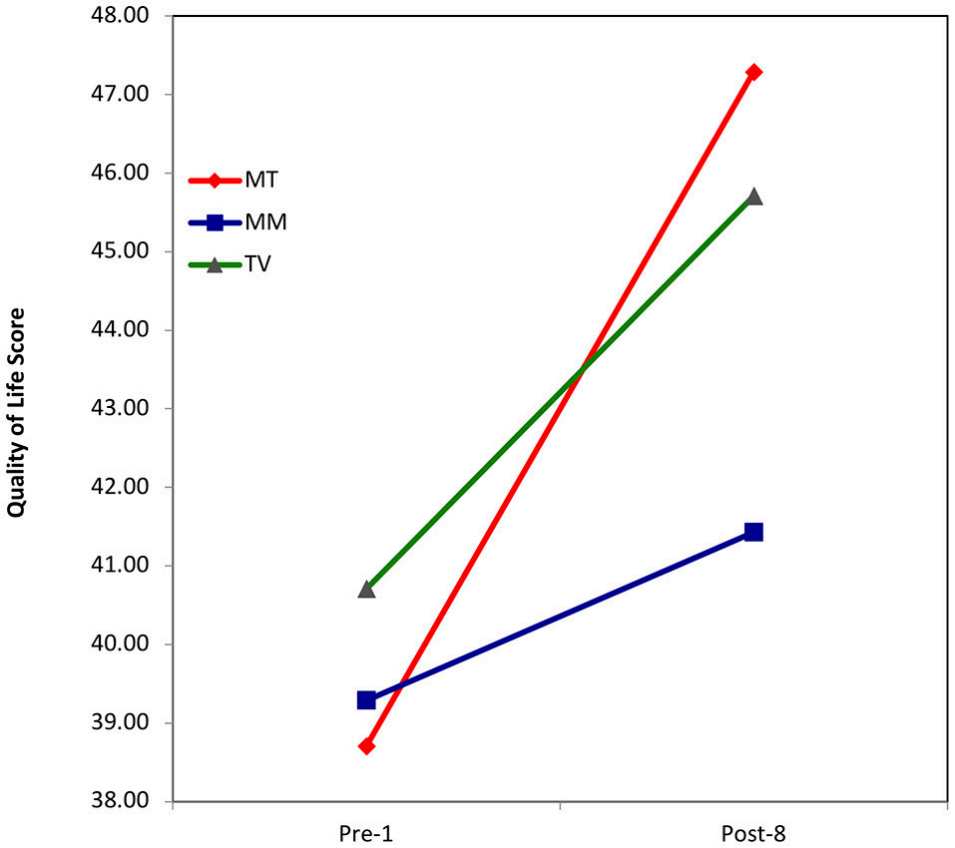
Quality of life score.

The plot of the interaction suggests that participants in the music medicine-listening group increased quality of life the least, while participants in both the music therapy-singing and the control-TV group increased more, with the biggest increase in the MT-singing group (See Figure 3).

### Positive and negative affect analysis

To determine the possible effects of a music therapy-singing, a music medicine-listening, or a control-TV group on affect of persons with dementia, a repeated measures ANOVA was conducted. Positive affect (PAS) and negative affect (NAS) were analyzed separately.

#### Positive affect analysis

The means and standard deviations of the positive affect score (PAS) are presented in Table 5.

**Table 5:** Means and standard deviations of total positive affect score.

	**Group**	**Mean**	**Standard deviation**	***N***
Pre 1	MT-singing	29.43	4.65	14
	MM-listening	28.21	6.20	14
	TV	28.75	8.61	8
	Total	28.81	6.12	36
Post 4	MT-singing	36.43	7.93	14
	MM-listening	29.07	7.18	14
	TV	25.38	9.32	8
	Total	31.11	8.97	36
Post 8	MT-singing	41.29	6.90	14
	MM-listening	31.00	4.71	14
	TV	27.38	9.09	8
	Total	34.19	8.79	36

*N, number of participants; MT, music therapy; MM, music medicine*.

A repeated-measures analysis of variance revealed that there was a significant difference between the pre- and post-sessions [*F*_(2, 33)_ = 6.68, *p* = 0.002], as well as a significant difference between groups [*F*_(2, 33)_ = 7.09, *p* = 0.003], at the *p* < 0.05 level. There was also a significant interaction [*F*_(4, 33)_ = 4.56, *p* = 0.001], as seen in Table 6.

**Table 6:** Repeated measures ANOVA for positive affect score.

**Source**	**Sum of squares**	**df**	**Mean square**	***F***	**Sig**.	**ηp2**
Group	1374.99	2	687.50	7.09	0.003^*^	0.300
Error Between	3202.19	33	97.04			
Pre-Post	340.13	2	170.06	6.68	0.002^*^	0.168
Interaction	571.59	4	142.89	5.61	0.001^*^	0.254
Error within	1680.06	33.00	50.91			

* *P < 0.05*.

To follow up the significant interaction to determine where the significant difference lies, a simple effects analysis was conducted. The three groups did not differ at the pre-test (*F* = 0.023, *p* = 0.977). However, at post-fourth session, there was a significant difference between MT-singing and TV group (*F* = 5.65, *p* = 0.010), whereas the difference between MT-singing vs. MM-listening group (*F* = 5.65, *p* = 0.051), or between TV vs. MM-listening group (*F* = 5.65, *p* = 0.553), was not significant. At post-eighth session, the differences between MT-singing and TV group (*F* = 13.55, *p* = 0.010), as well as between MT-singing and MM-listening group (*F* = 13.55, *p* = 0.001) were significant, whereas there was not a significant difference between TV and MM-listening group (*F* = 13.55, *p* = 0.450).

**Figure 4:**
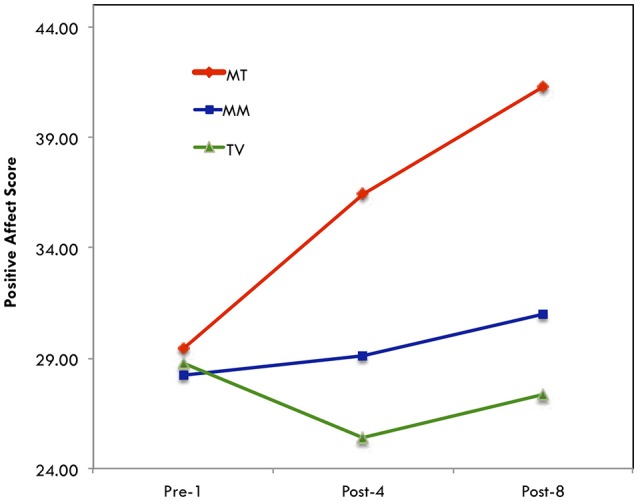
Positive affect score.

Because the sample size was somewhat small, a one-way, non-parametric analysis was conducted, using the Friedman Test, separately for each group. The analysis revealed that only the singing group changed in total positive affect scores significantly across time (see Table 7). The plot of the interaction also suggests that MT-singing group increased PAS (positive affect score) most, while MM-listening group and TV group remained flat (Figure 4).

**Table 7:** The Friedman test for positive affect score.

MT-singing	Chi Square = 15.53	*P* = 0.001^*^
MM-listening	Chi Square = 3.04	*P* = 0.219
TV	Chi Square = 3.17	*P* = 0.156

* *P < 0.05*.

#### Negative affect analysis

The means and standard deviations of negative affect score (NAS) are presented in Table 8.

**Table 8:** Means and standard deviations of negative affect score.

	**Group**	**Mean**	**Standard deviation**	***N***
Pre 1	MT-singing	14.86	3.96	14
	MM-listening	14.86	4.62	14
	TV	14.25	3.77	8
	Total	14.72	4.08	36
Post 4	MT-singing	10.86	1.02	14
	MM-listening	14.14	4.75	14
	TV	11.50	1.85	8
	Total	12.28	3.44	36
Post 8	MT-singing	10.07	0.27	14
	MM-listening	11.71	2.30	14
	TV	13.50	4.44	8
	Total	11.47	2.77	36

*MT, music therapy; MM, music medicine*.

A repeated-measures analysis of variance revealed that there was a significant effect for time, *F*_(2, 33)_ = 8.72, at the *p* < 0.05 level, as seen in Table 9. Since the interaction for negative affect score was not significant, it was not followed up. The differences between groups were not significant, either. Therefore, there were not any significant differences in the effects of MT-singing vs. MM-listening vs. TV group on total negative affect score.

**Table 9:** Repeated measures ANOVA for negative affect score.

**Source**	**Sum of squares**	**df**	**Mean square**	***F***	**Sig**.	**ηp2**
Group	58.75	2	29.38	1.95	0.158	–
Error Between	496.24	33	15.04			
Pre-Post	164.89	2	82.45	8.72	0.001^*^	0.209
Interaction	86.52	4	21.63	2.29	0.069	–
Error Within	623.91	66	9.45			

* *P < 0.05*.

**Figure 5:**
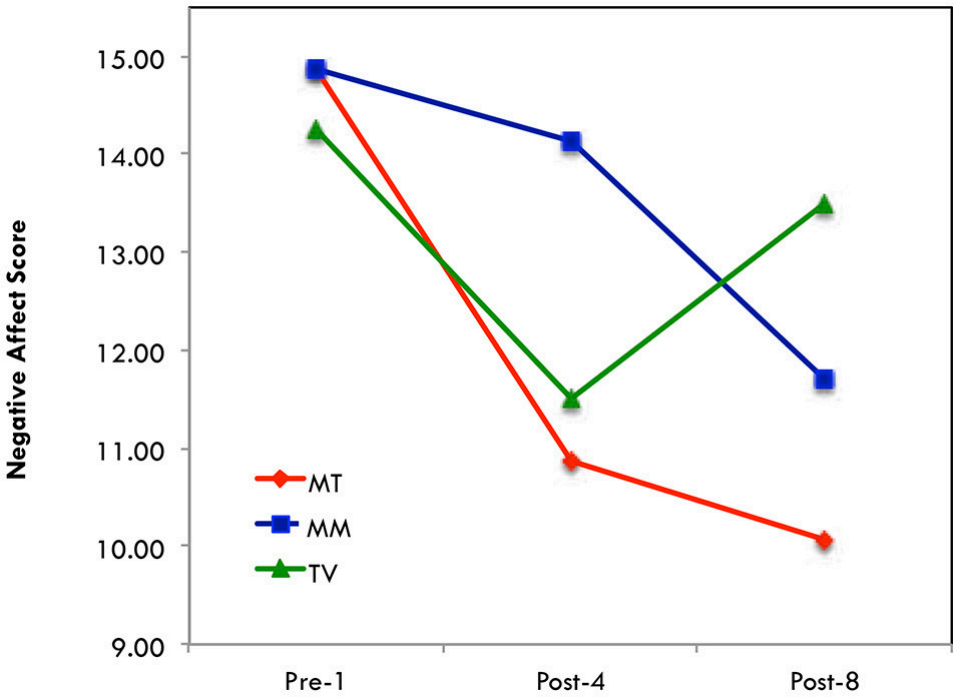
Negative affect score.

A one-way, non-parametric analysis was also conducted for NAS separately for each group due to somewhat small sample size and revealed that only the singing group's scores changed significantly across time (see Table 10). A visual examination of means is displayed in Figure 5, indicating that both the music therapy-singing and the music medicine-listening groups resulted in a decrease in negative affect.

**Table 10:** The Friedman test for negative affect score.

MT-Singing	Chi Square = 21.38	*p* = 0.001^*^
MM-Listening	Chi Square = 4.50	*p* = 0.105
TV	Chi Square = 4.56	*p* = 0.102

* *P < 0.05*.

## Discussion

The purpose of the current study was to compare the possible effects of a music therapy-singing group with those of a music medicine-listening group and a control-TV group on the quality of life and affect of persons with mild, moderate, and severe dementia living in a long-term care facility. The findings demonstrated that the short-term music therapy-singing group led by a music therapist had the larger effects on the quality of life and affect of persons with dementia, than the music medicine-listening group or the TV-control group. Due to the small number of participants and the short length of the intervention, the findings of the current study should be interpreted with caution.

### Quality of life

In the current study, only the music therapy-singing group improved the quality of life significantly from pre to posttest whereas the music medicine-listening group increased the least and the changes in both the music listening and the TV group were not significant. The larger effects of the music therapy-singing group on the quality of life when compared to those of the music medicine-listening group or the control-TV group may have been due to the therapeutic benefits of active singing, including the physical relaxation and social engagement. Since active singing requires deep breathing and increases oxygenation, it decreases muscle tension and promotes relaxation (22). Also, active group singing gives an opportunity for persons with dementia to interact with others and to experience a sense of a community, which becomes challenging due to the cognitive decline and behavioral changes (22). Since the QOL-AD evaluates one's perception on the physical condition, interpersonal relationships, and an ability to participate in meaningful activity, the physical and social benefits of the music therapy-singing group in the current study may have contributed to the significant improvements on the quality of life. Compared to the singing group, the music listening group and the TV group differed in that no real active interaction was required from the participants, and as a result, the participants in the music listening and the TV group may have been less motivated to participate or interact with each other.

The singing group in the current study was led by a music therapist with over 15 years of experience in dementia care with an emphasis on promoting a sense of self-worth, belonging and accomplishment through spontaneous expression and mutual interaction. The participants in the singing group of the current study were constantly encouraged and stimulated by the therapist to express and interact with the environment, including music, other participants, and the therapist. The music therapist carefully attuned to each individual's responses, assessed any strengths or potentials in the responses, and provided an opportunity to interact through the mutual musical process. Throughout the interactive process during singing, the participants may have experienced a sense of accomplishment and a sense of belonging, contributing to the significant improvements on the quality of life. Compared to the singing group, the music listening and the TV group in the current study were implemented by activity staff who were instructed to validate and process the participants' responses. However, they did not have the same level of training as the music therapist, especially in facilitating a group process. As a result, the mutual interaction between the participants and a facilitator in the music listening or the TV group may have not been as active as in the music therapy-singing group.

It is interesting to note that all three groups, including the music medicine-listening group and the control-TV group, demonstrated improvements in quality of life at the end of a total of eight interventions, even though the changes in the music listening and the TV group were not significant. It is probable that a high percentage of persons with dementia in long-term care settings may be exposed to passive music listening or TV watching frequently at an individual level. However, the music listening and the TV interventions in the current study were implemented in a group format in the presence of activity staff, who was instructed to validate and process the participants' responses. Even though the level of engagement may not have been as active as in the music therapy-singing group, the participants in the music listening and the TV group in the current study may have experienced higher level of participation and involvement than their daily routine. The findings of the current study that quality of life improved in all three groups may demonstrate the importance of engaging persons with dementia in a residential setting. Further investigation is needed to determine the possible effects of an engagement of the persons with dementia in meaningful activities on quality of life.

There are a small number of music therapy studies that investigated the effects of music therapy on quality of life of persons with dementia, and the findings are not consistent. Ridder et al. (17) found that quality of life was increased after individual music therapy with various music interventions, whereas it was decreased during standard care, without a significant difference. Ridder et al. (18) also reported that individual music therapy with therapeutic singing showed an improvement of quality of life of an individual with dementia. However, another participant's total score of quality of life was decreased and it was argued that the decrease in the quality of life score in this case should be interpreted positively, because the individual seemed more relaxed and calmer, thus meeting her therapeutic needs to relax and calm down. Raglio et al. (16) found that individual music therapy with active improvisation improved quality of life, whereas individualized music listening worsened it, without significant differences in both changes. Solé et al. (20) found that there was no significant improvement in quality of life scores after 12 weekly group music therapy with various music interventions, including singing. It was speculated that the measurement tool used in the study, may not have reflected the effects of music therapy appropriately. It is noted that different standardized measurement tools were utilized to assess quality of life in those studies, including the Alzheimer's Disease-Related Quality of Life (17, 18), the Cornell-Brown Scale for Quality of Life (16), and the GENCAT for Quality of Life (20), which may have contributed to the inconsistent findings.

The findings of the current study regarding the significant effects of group singing are in contrast with a couple of studies by non-music therapists utilizing singing for persons of dementia. Särkämö et al. (19) compared the effects of singing with those of listening on quality of life of persons with dementia and reported that music listening was found to have a more positive long-term effect on quality of life than singing. However, singing in the study of Särkämö et al. (19) was utilized in a different setting with different focuses from the current study. Singing in the study of Särkämö et al. (19) was facilitated by a trained music educator with the purpose of coaching caregivers so that they can apply singing as a part of everyday leisure activity outside the therapeutic setting. On the other hand, the listening group in the study of Särkämö et al. (19) was led by a music therapist and consisted of listening to a CD and discussing emotions, thoughts, and memories, which may have given more opportunity for the participants to communicate and interact with each other than during the singing. The researchers did not have a control over the frequency of the music intervention delivery by caregivers during the 6-month follow-up period and caregivers may have provided listening more often than singing during the 6-month follow-up period because it was probably easier for them to implement listening than singing. Cooke et al. (15) also found that a live group music program with singing was less effective than a reading group in improving the quality of life. The music program was led by a musician and consisted of a 30-min of musician-led singing and a 10-min of pre-recorded instrumental music listening. The music program was very structured, following a set pattern to ensure treatment consistency. In contrast, the reading group, which was led by a trained research assistant, included a range of activities, such as quizzes and discussions about the past, which may have stimulated greater involvement and interaction from the participants than the singing group. The different effects of the singing led by a music educator or a musician on quality of life in the study of Särkämö et al. (19) and Cooke et al. (15) may have been due to the different training and approaches of the facilitators, which may have contributed to the different level of interaction and engagement of the participants during singing. The role of the facilitator of music interventions on the therapeutic outcome needs to be explored further.

### Affect

The music therapy-singing group of the current study improved positive affect significantly between the baseline and post-sessions, whereas a slight increase of positive affect was found in the music medicine-listening group and a decrease of positive affect was found in the control-TV group without any statistical significance. Regarding negative affect, both the music therapy-singing and the music medicine-listening group decreased across time, and the difference between the baseline and post-sessions was significant in the singing group only, whereas the TV group demonstrated an increase of negative affect between the post-fourth and the post-eighth session.

The neurological benefits of singing may have contributed to the larger effects of the singing group on improving positive affect and decreasing negative affect than those of listening group. Unlike the music listening, singing places additional demands on the nervous system, creating a strong coupling of perception and action (50). Group singing is also found to release endorphins, a hormone which is associated with feeling of pleasure, as well as oxytocin, another hormone which is associated with alleviating anxiety and stress (51).

The participants' preferences for the TV programs in the control-TV group were not assessed or reflected, whereas music programs for singing and listening group were created based on the participants' preferred music and musicians. The lack of reflecting the participants' preference in the choice of the TV group may have contributed to the decrease of positive affect and the increase of negative affect, as well as the highest drop-out rate of participants in the TV group. Out of 17 participants who were assigned to the TV group, nine dropped out over the course of the study, and only eight completed the intervention. The possible effects of accommodating the participants' preference on the affect need to be explored further.

### Limitations

In the current study, a complete blinding for the participant's group assignment to the assessors was not guaranteed because the schedule of interventions for the current study was incorporated into the facility's daily activity schedule and displayed in the public areas throughout the facility on a daily basis. Furthermore, the participants' group assignments became obvious to the assessors because they conducted an interview with the participants right before and after specific intervention sessions. As a result, it was impossible to blind assessors to the participants' group allocation.

At the initial phase of the current study, 89 participants who met the criteria of the current study were identified. However, the final sample size was relatively small. Of the 89 eligible participants, 37 (42%) refused and only 52 (58%) agreed to participate. Of the 52 participants, only 37 completed the assigned interventions. This may be due to the vulnerable state of the participants with advanced age and may reflect the reality of most long-term care settings. A future research studies utilizing multiple settings to recruit potential participants may increase the probability of a larger sample size.

The current study relied entirely on the participants' self-reports to determine the effectiveness of interventions, and the accuracy and validity of data from the participants with dementia at varying degrees may be questioned. Most research studies in dementia care rely on either family or professional caregivers' observations, instead of self-reports of persons with dementia because memory, language and insight of persons with dementia are impaired to varying degrees depending on the extent of the dementia process (11). However, the indirect approach may not be a true reflection of persons with dementia, whose perception may be different from that of their caregivers (52). A self-report can be the most desirable source of data especially for subjective outcomes, such as emotions and quality of life which may be relatively less affected by the dementia process compared to the cognitive functioning (53). The attempt of the current study to directly reflect the subjective perception of persons with dementia on the quality of life and affect may be of importance, despite the potential danger of bias of self-report.

### Implications for clinical practice

The number and proportion of persons with dementia in long-term care settings are rapidly increasing and there are a great deal of interest and a growing demand for effective interventions for persons with dementia. Music interventions have been widely used for persons with dementia in long-term care settings by both music therapists and other healthcare professionals, and there are various levels of music and music therapy interventions. The possible effects of different levels of music therapy and music medicine practices in dementia care need to be further examined and communicated. At the same time, music therapist professionals need to recognize their critical role in music medicine practice in dementia care and take an active role to provide the professional guidance through education and training medical personnel to improve the implementation of music medicine practice, as well as to promote the expansion of music therapy practice for persons with dementia. Based on the findings of the current study, active singing group with the therapeutic goals of improving a sense of self-worth, belonging and accomplishment for persons of dementia in long-term care settings need to be promoted. Furthermore, it may be appropriate for music therapists to encourage and train professional and family caregivers to utilize active singing to improve daily caregiving with persons with dementia.

### Implications for future research

With the focus on the evidence-based practice in modern health care, it is critical to add more studies with high quality to the music therapy research for persons with dementia. The current study attempted to improve the quality of research by adopting a randomized controlled trial design and aiming for a larger sample size. However, due to the vulnerability of the participants with an advanced age, the final sample size turned out to be relatively small. Future research studies need to continue to aim for high quality by using a randomized controlled trial design and a larger sample size, as well as blinding assessors.

The outcomes of the current study need to be explored further to determine which factors contributed to the significant effects of the music therapy-singing group on the quality of life and affect of persons with dementia. Was the significant effects of music therapy-singing group on the quality of life and affect due to the active nature of singing or the therapeutic process of enhancing a sense of self-worth, belonging and accomplishment? Any difference between a therapist-led singing group and a therapist-led listening group or between a therapist-led singing group and a non-music therapist-led singing group needs to be further investigated. The possible effects of the personal or musical characteristics of the therapist on the therapeutic outcomes also need to be explored. Furthermore, the effectiveness of other active methods of music therapy, such as improvisation, needs to be examined and compared.

## Conclusion

Persons with dementia often experience a wide range of discouraging changes and impairments due to the progressive and degenerative nature of dementia, which significantly contribute to the reduced quality of life and emotional well-being. The number and proportion of persons with dementia in long-term care settings are rapidly increasing and improving the quality of life and psychological well-being of persons with dementia is a high priority of healthcare professionals. Based on the findings of the current study, a music-therapy singing group with an emphasis on promoting a sense of self-worth, belonging and accomplishment through expression and mutual interaction is recommended as a valid intervention to improve quality of life and affect of persons with dementia. Furthermore, the possible effects of various aspects of music applications on the clinical areas of dementia care need to be tested and communicated.

## Author contributions

The author confirms being the sole contributor of this work and has approved it for publication.

### Conflict of interest statement

The author declares that the research was conducted in the absence of any commercial or financial relationships that could be construed as a potential conflict of interest.
